# Visible-light photoredox catalyzed synthesis of pyrroloisoquinolines via organocatalytic oxidation/[3 + 2] cycloaddition/oxidative aromatization reaction cascade with Rose Bengal

**DOI:** 10.3762/bjoc.10.122

**Published:** 2014-05-27

**Authors:** Carlos Vila, Jonathan Lau, Magnus Rueping

**Affiliations:** 1 Institute of Organic Chemistry, RWTH Aachen University, Landoltweg 1, D-52074 Aachen, Germany

**Keywords:** alkaloids, [3 + 2] cycloaddition, organocatalysis, oxidation, photochemistry, photoredox catalysis, Rose Bengal, visible-light

## Abstract

Pyrrolo[2,1-*a*]isoquinoline alkaloids have been prepared via a visible light photoredox catalyzed oxidation/[3 + 2] cycloaddition/oxidative aromatization cascade using Rose Bengal as an organo-photocatalyst. A variety of pyrroloisoquinolines have been obtained in good yields under mild and metal-free reaction conditions.

## Introduction

**Figure 1:**
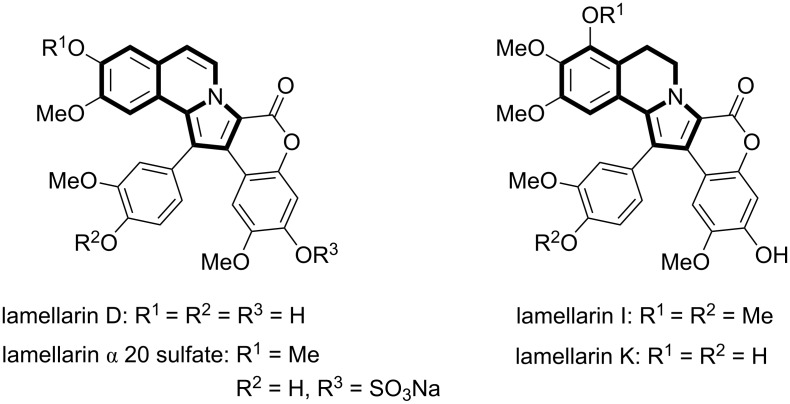
Representative examples of lamellarin alkaloids.

Pyrrolo[2,1-*a*]isoquinolines constitute the core structure of the natural products family lamellarin alkaloids (Figure 1) [1–4]. These alkaloids display numerous biological activities such as inhibitor of human topoisomerase I by lamellarin D [5] or inhibition of HIV integrase by lamellarin α*-*20-sulfate [6–7]. Moreover lamellarin I and lamellarin K also showed potential antitumor activities [8–9]. Due to their potential biological activities, the synthesis of pyrrolo[2,1-*a*]isoquinolines has become a very interesting, important and attractive goal in organic synthesis [10–20]. For example, dipolar [3 + 2] cycloaddition using azomethine ylides [21] is a powerful class of reactions that permits the synthesis of structural complex molecules in a straightforward way and has been used for the efficient synthesis of this type of compounds [22–26]. Recently, several metal mediated syntheses using a [3 + 2] cycloaddition reaction have been described in the literature. Porco Jr. et al. [27] described a silver-catalyzed cycloisomerization/dipolar cycloaddition for the synthesis of the pyrrolo[2,1-*a*]isoquinolines. Wang and co-workers described a copper catalyzed oxidation/[3 + 2] cycloaddition/aromatization cascade [28]. Also, Xiao disclosed a very elegant oxidation/[3 + 2] cycloaddition/aromatization cascade catalyzed by [Ru(bpy)_3_]^3+^ under irradiation with visible light [29]. In this context, very recently Zhao reported the same reaction using C_60_-Bodipy hybrids [30] and porous material immobilized iodo-Bodipy [31] as photocatalysts, obtaining in both cases good yields for different pyrrolo[2,1-*a*]isoquinolines. Finally, Lu presented in 2013 a dirhodium complex for the synthesis of these compounds [32]. Despite these elegant and important syntheses of pyrrolo[2,1-*a*]isoquinolines through dipolar [3 + 2] cycloaddition, the development of metal-free syntheses using visible light photoredox catalysis with simple organic dyes remained unexplored. Visible-light photoredox catalysis has emerged as an important field and has attracted increasing attention in recent years [33–42]. Thus, in the last years spectacular advances in visible-light photoredox catalysis have been made and this kind of catalysis has become a powerful tool in organic synthesis. In this context, the use of organic dyes as photoredox catalysts [40–42] has been demonstrated by several groups [43–61] and became a useful alternative to the inorganic photoredox catalysts that are expensive and sometimes toxic. The organic dyes have very important qualities such as being inexpensive, environmentally friendly and easy to handle. As a part of our ongoing research on photoredox catalysis [62–72], we herein present a synthesis of pyrrolo[2,1-*a*]isoquinolines through an oxidation/[3+2] cycloaddition/aromatization cascade catalyzed by Rose Bengal under irradiation with green LEDs.

## Results and Discussion

**Scheme 1:**
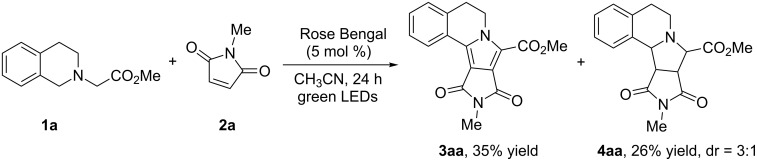
Photocatalytic metal free construction of pyrrolo[2,1-*a*]isoquinolines.

Initially, we focused on the reaction between methyl dihydroisoquinoline ester **1a** and *N*-methylmaleimide (**2a**) catalyzed by Rose Bengal. Although the [3 + 2] cycloaddition occurs smoothly in the presence of Rose Bengal (5 mol %) in acetonitrile under irradiation with visible light, the reaction was not selective affording the dihydropyrrolo[2,1-*a*]isoquinoline **3aa** in 35% yield and the hexahydropyrrolo[2,1-*a*]isoquinoline **4aa** in 26% yield, after column chromatography (Scheme 1).

In order to improve the selectivity of the reaction to the aromatized product **3aa**, *N*-bromosuccinimide was added to the reaction mixture when the starting materials were completely consumed [29–31,73]. In this case the desired product **3aa** was obtained in 72% yield (Table 1, entry 1). Other organic dyes such as Rhodamine B or Eosin Y were less efficient compared to Rose Bengal (Table 1, entries 2 and 3, respectively). Several solvents were tested without an improvement in the yield of the product (Table 1, entries 4–9). Finally, after tuning the relative amounts of the reagents, the product **3aa** was isolated in 76% yield (Table 1, entry 12).

**Table 1:** Optimization of the reaction conditions.^a^

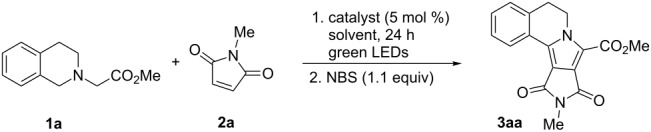			

Entry	Catalyst	Solvent	Yield (%)^b^

1	Rose Bengal	CH_3_CN	72
2	Rhodamine B	CH_3_CN	11
3	Eosin Y	CH_3_CN	40
4	Rose Bengal	THF	29
5	Rose Bengal	CH_2_Cl_2_	26
6	Rose Bengal	toluene	14
7	Rose Bengal	DMF	65
8	Rose Bengal	MeOH	52
9	Rose Bengal	EtOAc	16
10^c^	Rose Bengal	CH_3_CN	64
11^d^	Rose Bengal	CH_3_CN	60
12^e^	Rose Bengal	CH_3_CN	76

^a^Reaction conditions: **1a** (0.2 mmol), **2a** (0.2 mmol), organic dye (5 mol %), solvent (1 mL), green LEDs irradiation for 24 hours. NBS (1.1 equiv) was added to the reaction mixture and stirring was continued for 1 hour. ^b^Yields of the isolated products after column chromatography. ^c^1.25 equiv of **1a** was used. ^d^1.25 equiv of **2a** was used. ^e^1.1 equiv of **1a** was used.

**Scheme 2:**
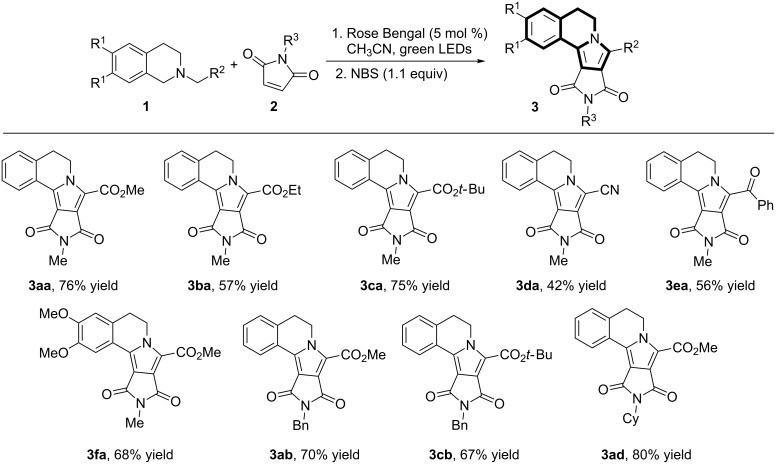
Evaluation of the substrate scope.

With the optimal conditions in hand, we examined the substrate scope for the photoreaction catalyzed by Rose Bengal (Scheme 2). Various tetrahydroisoquinolines with different electron-withdrawing groups (R^2^) such as methyl ester (**1a**), ethyl ester (**1b**), *tert*-butyl ester (**1c**), cyano (**1d**) or aromatic ketone (**1e**) were reacted with *N*-methylmaleimide (**2a**) and gave the corresponding products **3** in moderate to good yields. In addition, different *N*-substituted maleimides were tested under the optimized reaction conditions to give the corresponding products with good yields. Incorporation of methoxy groups at C-6 and C-7 in the dihydroisoquinoline core was well tolerated, affording the corresponding product **3fa** in 68% yield.

**Scheme 3:**
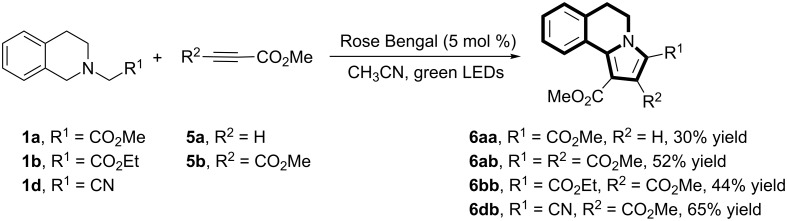
Evaluation of the substrate scope with activated alkynes.

To demonstrate the synthetic utility of the oxidation/[3 + 2] cycloaddition/aromatization cascade we examined other dipolarophiles such as activated alkynes **5**. In this case, the addition of NBS was not necessary, and the corresponding products **6** were isolated in moderate yields (Scheme 3).

## Conclusion

In conclusion, we have developed a metal-free photoredox oxidation/[3 + 2] dipolar cycloaddition/oxidative aromatization cascade catalyzed by Rose Bengal using visible-light. This protocol offers a “green” and straightforward synthesis of pyrrolo[2,1-*a*]isoquinolines starting from readily available maleimides and tetrahydroisoquinolines. Further investigations to expand the scope and potential of this methodology are underway in our laboratory.

## Supporting Information

File 1 Experimental details and characterization of the synthesized compounds.

## References

[1] Bailly C (2004). Curr Med Chem: Anti-Cancer Agents.

[2] Handy S T, Zhang Y (2005). Org Prep Proced Int.

[3] Fan H, Peng J, Hamann M T, Hu J-F (2008). Chem Rev.

[4] Pla D, Albericio F, Alvarez M (2008). Anti-Cancer Agents Med Chem.

[5] Marco E, Laine W, Tardy C, Lansiaux A, Iwao M, Ishibashi F, Bailly C, Gago F (2005). J Med Chem.

[6] Reddy M V R, Rao M R, Rhodes D, Hansen M S T, Rubins K, Bushman F D, Venkateswarlu Y, Faulkner D (1999). J Med Chem.

[7] Aubry A, Pan X-S, Fisher L M, Jarlier V, Cambau E (2004). Antimicrob Agents Chemother.

[8] Reddy S M, Srinivasulu M, Satanarayana N, Kondapi A K, Venkateswarlu Y (2005). Tetrahedron.

[9] Quesada A R, Garcia Grávalos M D, Fernández Puentes J L (1996). Br J Cancer.

[10] Heim A, Terpin A, Steglich W (1997). Angew Chem, Int Ed Engl.

[11] Ploypradith P, Mahidol C, Sahakitpichan P, Wongbundit S, Ruchirawat S (2004). Angew Chem, Int Ed.

[12] Boger D L, Boyce C W, Labroli M A, Sehon C A, Jin Q (1999). J Am Chem Soc.

[13] Banwell M G, Flynn B L, Stewart S G (1998). J Org Chem.

[14] Handy S T, Zhang Y, Bregman H (2004). J Org Chem.

[15] Ploypradith P, Kagan R K, Ruchirawat S (2005). J Org Chem.

[16] Ohta T, Fukuda T, Ishibashi F, Iwao M (2009). J Org Chem.

[17] Gupton J T, Clough S C, Miller R B, Lukens J R, Henry C A, Kanters R P F, Sikorski J A (2003). Tetrahedron.

[18] Fujikawa N, Ohta T, Yamaguchi T, Fukuda T, Ishibashi F, Iwao M (2006). Tetrahedron.

[19] Chen L, Xu M-H (2009). Adv Synth Catal.

[20] Yadav J S, Gayathri K U, Reddy B V S, Prasad A R (2009). Synlett.

[21] Najera C, Sansano J M (1998). Curr Org Chem.

[22] Ishibashi F, Miyazaki Y, Iwao M (1997). Tetrahedron.

[23] Banwell M G, Flynn B L, Hockless D C R (1997). Chem Commun.

[24] Cironi P, Manzanares I, Albericio F, Álvarez M (2003). Org Lett.

[25] Ploypradith P, Petchmanee T, Sahakitpichan P, Litvinas N D, Ruchirawat S (2006). J Org Chem.

[26] Grigg R, Heaney F (1989). J Chem Soc, Perkin Trans 1.

[27] Su S, Porco J A (2007). J Am Chem Soc.

[28] Yu C, Zhang Y, Zhang S, Li H, Wang W (2011). Chem Commun.

[29] Zou Y-Q, Lu L-Q, Fu L, Chang N-J, Rong J, Chen J-R, Xiao W-J (2011). Angew Chem, Int Ed.

[30] Huang L, Zhao J (2013). Chem Commun.

[31] Guo S, Zhang H, Huang L, Guo Z, Xiong G, Zhao J (2013). Chem Commun.

[32] Wang H-T, Lu C-D (2013). Tetrahedron Lett.

[33] Zeitler K (2009). Angew Chem, Int Ed.

[34] Yoon T P, Ischay M A, Du J (2010). Nat Chem.

[35] Narayanam J M R, Stephenson C R J (2011). Chem Soc Rev.

[36] Xuan J, Xiao W-J (2012). Angew Chem, Int Ed.

[37] Shi L, Xia W (2012). Chem Soc Rev.

[38] Prier C K, Rankic D A, MacMillan D W C (2013). Chem Rev.

[39] Hu J, Wang J, Nguyen T H, Zheng N (2013). Beilstein J Org Chem.

[40] Ravelli D, Fagnoni M (2012). ChemCatChem.

[41] Ravelli D, Fagnoni M, Albini A (2013). Chem Soc Rev.

[42] Nicewicz D C, Nguyen T M (2014). ACS Catal.

[43] Liu H, Feng W, Kee C W, Zhao Y, Leow D, Pan Y, Tan C-H (2010). Green Chem.

[44] Pan Y, Kee C W, Chen L, Tan C-H (2011). Green Chem.

[45] Pan Y, Wang S, Kee C W, Dubuisson E, Yang Y, Loh K P, Tan C-H (2011). Green Chem.

[46] Neumann M, Füldner S, König B, Zeitler K (2011). Angew Chem, Int Ed.

[47] Hari D P, König B (2011). Org Lett.

[48] Liu Q, Li Y-N, Zhang H-H, Chen B, Tung C-H, Wu L-Z (2012). Chem–Eur J.

[49] Fidaly K, Ceballos C, Falguières A, Veitia M S-I, Guy A, Ferroud C (2012). Green Chem.

[50] Fu W, Guo W, Zou G, Xu C (2012). J Fluorine Chem.

[51] Hari D P, Schroll P, König B (2012). J Am Chem Soc.

[52] Hari D P, Hering T, König B (2012). Org Lett.

[53] Neumann M, Zeitler K (2012). Org Lett.

[54] Hamilton D S, Nicewicz D A (2012). J Am Chem Soc.

[55] Rueping M, Vila C, Bootwicha T (2013). ACS Catal.

[56] Grandjean J, Nicewicz D A (2013). Angew Chem, Int Ed.

[57] Riener M, Nicewicz D A (2013). Chem Sci.

[58] Wilger D J, Gesmundo N J, Nicewicz D A (2013). Chem Sci.

[59] Nguyen T M, Nicewicz D A (2013). J Am Chem Soc.

[60] Perkowski A J, Nicewicz D A (2013). J Am Chem Soc.

[61] Pitre S P, McTiernan C D, Ismaili H, Scaiano J C (2013). J Am Chem Soc.

[62] Rueping M, Vila C, Koenings R M, Poscharny K, Fabry D C (2011). Chem Commun.

[63] Rueping M, Zhu S, Koenings R M (2011). Chem Commun.

[64] Rueping M, Leonori D, Poisson T (2011). Chem Commun.

[65] Rueping M, Zhu S, Koenings R M (2011). Chem Commun.

[66] Rueping M, Zoller J, Fabry D C, Poscharny K, Koenings R M, Weirich T E, Mayer J (2012). Chem–Eur J.

[67] Rueping M, Koenings R M, Poscharny K, Fabry D C, Leonori D, Vila C (2012). Chem–Eur J.

[68] Rueping M, Vila C, Szadkowska A, Koenigs R M, Fronert J (2012). ACS Catal.

[69] Zhu S, Rueping M (2012). Chem Commun.

[70] Zhu S, Das A, Bui L, Zhou H, Curran D P, Rueping M (2013). J Am Chem Soc.

[71] Rueping M, Vila C (2013). Org Lett.

[72] Vila C, Rueping M (2013). Green Chem.

[73] Tóth J, Váradi L, Dancsó A, Blaskó G, Töke L, Nyerges M (2007). Synlett.

