# Supplementary material for: Visible-light photoredox catalyzed synthesis of pyrroloisoquinolines via organocatalytic oxidation/[3 + 2] cycloaddition/oxidative aromatization reaction cascade with Rose Bengal
Source: Beilstein J Org Chem. 2014 May 27;10:1233–8. doi: 10.3762/bjoc.10.122 (PMC4077507; doi:10.3762/bjoc.10.122)
Supplement: File 1 — Experimental details and characterization of the synthesized compounds. [file Beilstein_J_Org_Chem-10-1233-s001.pdf]

Supporting Information

for

Visible-light photoredox catalyzed synthesis of pyrroloisoquinolines via organocatalytic oxidation/[3 + 2] cycloaddition/oxidative aromatization reaction cascade with Rose Bengal

Carlos Vila, Jonathan Lau and Magnus Rueping*

Address: Institute of Organic Chemistry, RWTH Aachen University,

Landoltweg 1, D-52074 Aachen, Germany

Email: Magnus Rueping* - magnus.rueping@rwth-aachen.de

* Corresponding author

Experimental details and characterization of the synthesized compounds

Table of Contents

General methods S2

General procedures for synthesis of compounds **3** S3

Characterization of products **3** S3

General procedure for synthesis of compound **6** S6

Characterization of product **6** S6

NMR spectra of compounds **3** and **6** S8

General methods. Unless otherwise noted, all commercially available compounds were used as received. Analytical grade solvents used for reaction were dried and distilled prior to use. Analytical thin-layer chromatography (TLC) was performed on Merck silica gel 60 aluminium plates with F-254 indicator, visualised by UV irradiation. Column chromatography was performed using MN silica gel (particle size 0.040–0.063 mm). ^{1}H NMR and ^{13}C NMR were recorded on a Mercury 300, Inova 400 or VNMRS-600 spectrometer in $CDCl_3$ or toluene-d_8 with residual proton signal of the deuterated solvents as the internal reference (δ_H = 7.26 ppm and δ_C = 77.00 ppm for $CDCl_3$) or TMS as internal reference. Data are reported in the following order: chemical shift (δ) in ppm; multiplicities are indicated s (singlet), d (doublet), t (triplet), q (quartet), dd (doublet of doublets), td (triplet of doublets), tt (triplet of triplets), ddd (doublet of doublet of doublets), m (multiplet), p (pentet), s (sextet); coupling constants (*J*) are in Hertz (Hz). ^{13}C NMR spectra were acquired on a broad band decoupled mode. Mass spectra was conducted on GC–MS Shimadzu QP2010 (column: Equity®-5, length × I.D. 30 m × 0.25 mm, d_f 0.25 μm, lot # 28089-U, Supelco). IR spectra were measured in a Perkin-Elmer ATR apparatus and are reported in terms of frequency of absorption (cm^{-1}).

General procedure A for synthesis of compounds 3

In a vial, Rose Bengal (10.2 mg, 0.01 mmol), amine **1** (0.22 mmol) and *N*-alkylmaleimide **2** (0.20 mmol) were dissolved in 1 mL acetonitrile. The reaction mixture was stirred for 24–48 hours under irradiation with 1 W green LED 30 cm strip (distance app. 5 cm) until the starting material was consumed. Once the starting material was consumed, *N*-bromosuccinimide (0.22 mmol) was added and stirred for 1 hour. After the solvent was removed under reduced pressure and the crude reaction mixture was directly charged on silica gel and purified by column chromatography (hexane/EtOAc 95:5 to 9:1) to afford the corresponding product **3**.

Characterization of products 3

Methyl 10-methyl-9,11-dioxo-6,9,10,11-tetrahydro-5*H*-pyrrolo[3',4':3,4]pyrrolo[2,1-*a*] isoquinoline-8-carboxylate (3aa)

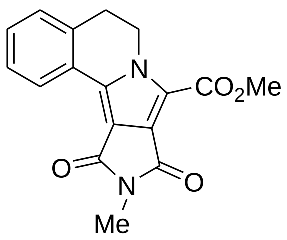

Synthesized according to the general procedure **A**; m.p.: 195-197 °C. ^{1}H NMR (400 MHz, $CDCl_3$): *δ* 8.49 (dd, *J* = 7.6, 1.3 Hz, 1H), 7.40 (dt, *J* = 7.6, 1.4 Hz, 1H), 7.35 (dt, *J* = 7.4, 1.5 Hz, 1H), 7.25 (d, *J* = 6.2 Hz, 1H), 4.69 (t, *J* = 6.9 Hz, 2H), 3.96 (s, 3H), 3.14 (t, *J* = 6.9 Hz, 2H), 3.09 (s, 3H) ppm; ^{13}C NMR (100 MHz, $CDCl_3$) *δ* 164.1, 162.8, 160.1, 133.0, 132.3, 130.2, 128.0, 127.8, 127.6, 125.9, 125.6, 117.6, 116.7, 52.3, 43.3, 28.3, 24.2 ppm; IR (KBr): ν = 3435, 2955, 1752, 1698, 1474, 1427, 1273, 1190, 978, 735, 688 cm^{-1}; MS-EI: *m/z* (%) 310 (M^+, 100), 278 (23), 277 (36).

Ethyl 10-methyl-9,11-dioxo-6,9,10,11-tetrahydro-5*H*-pyrrolo[3',4':3,4]pyrrolo[2,1-*a*] isoquinoline-8-carboxylate (3ba)[1]

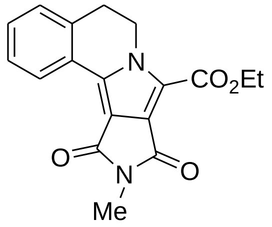

Synthesized according to the general procedure **A**; ^{1}H NMR (400 MHz, $CDCl_3$): *δ* 8.48 (dd, *J* = 7.6, 1.2 Hz, 1H), 7.38 (dt, *J* = 7.5, 1.5 Hz, 1H), 7.34 (dt, *J* = 7.4, 1.6 Hz, 1H), 7.24 (dd, *J* = 7.3, 0.9 Hz, 1H), 4.68 (dd, *J* = 7.3, 6.6 Hz, 2H), 4.39 (q, *J* = 7.1 Hz, 2H), 3.12 (t, *J* = 6.9 Hz, 2H), 3.08 (s, 3H), 1.46 (t, *J* = 7.1 Hz, 3H) ppm; ^{13}C NMR (100 MHz, $CDCl_3$) *δ* 164.1, 162.8, 159.6, 132.8, 132.3, 130.1, 127.9, 127.8, 127.6, 125.8, 125.6, 118.1, 116.6, 61.5, 43.2, 28.3, 24.2, 14.2 ppm;

tert-Butyl 10-methyl-9,11-dioxo-6,9,10,11-tetrahydro-5*H*-pyrrolo[3',4':3,4]pyrrolo[2,1-*a*] isoquinoline-8-carboxylate (3ca)

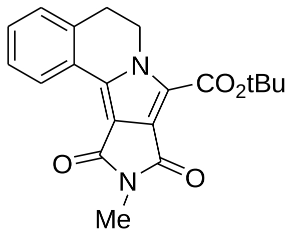

Synthesized according to the general procedure **A**; m.p.: 170-173 °C. ^{1}H NMR (400 MHz, $CDCl_3$): δ 8.47 (dd, *J* = 7.6, 0.9 Hz, 1H), 7.37 (dt, *J* = 7.5, 1.4 Hz, 1H), 7.32 (dt, *J* = 7.4, 1.5 Hz, 1H), 7.23 (dd, *J* = 7.4, 0.6 Hz, 1H), 4.66 (t, *J* = 6.8 Hz, 2H), 3.12-3.08 (m, 5H), 1.64 (s, 9H) ppm; ^{13}C NMR (100 MHz, $CDCl_3$) δ 164.2, 162.9, 159.0, 132.3, 132.3, 129.9, 127.9, 127.7, 127.5, 125.7, 125.2, 119.6, 116.4, 83.1, 43.1, 28.3, 28.3, 24.2 ppm; IR (KBr): ν = 2973, 1757, 1694, 1552, 1473, 1422, 1363, 1286, 1144, 970, 853, 735, 676 cm^{-1}; MS-EI: *m/z* (%) 352 (M^+, 57), 296 (100), 278 (31), 255 (55), 252 (61), 208 (52).

10-Methyl-9,11-dioxo-6,9,10,11-tetrahydro-5*H*-pyrrolo[3',4':3,4]pyrrolo[2,1-*a*] isoquinoline-8-carbonitrile (3da)

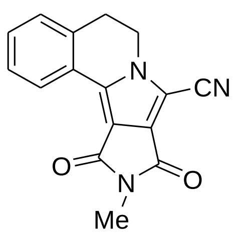

Synthesized according to the general procedure **A**; m.p.: 250-253°C. ^{1}H NMR (400 MHz, $CDCl_3$): δ 8.40 (dd, *J* = 7.4, 1.6 Hz, 1H), 7.44-7.37 (m, 2H), 7.29 (dd, *J* = 7.0, 1.3 Hz, 1H), 4.31 (t, *J* = 7.0 Hz, 2H), 3.23 (t, *J* = 6.9 Hz, 2H), 3.10 (s, 3H) ppm; ^{13}C NMR (100 MHz, $CDCl_3$) δ 163.1, 161.7, 133.0, 131.7, 130.9, 128.8, 128.3, 128.0, 127.8, 124.8, 116.9, 109.9, 98.6, 43.9, 28.0, 24.4 ppm; IR (KBr): ν = 2928, 2226, 1756, 1692, 1551, 1472, 1422, 1351, 1234, 1028, 976, 773, 736 cm^{-1}; MS-EI: *m/z* (%) 277 (M^+, 100), 233 (71), 164 (20).

(10-Methyl-9,11-dioxo-6,9,10,11-tetrahydro-5*H*-pyrrolo[3',4':3,4]pyrrolo[2,1-*a*]isoquin-3-yl)-(phenyl)-methanone (3ea)

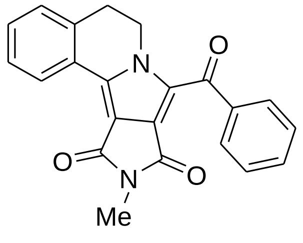

Synthesized according to the general procedure **A**; m.p.: 205-208 °C. ^{1}H NMR (400 MHz, $CDCl_3$): δ 8.55 (dd, *J* = 7.6, 1.2 Hz, 1H), 7.92 (dd, *J* = 8.4, 1.3 Hz, 2H), 7.66 (tt, *J* = 7.5, 1.4 Hz, 1H), 7.50 (t, *J* = 7.7, 2H), 7.42 (dt, *J* = 7.5, 1.5, 1H), 7.37 (dt, *J* = 7.4, 1.5, 1H), 7.28 (dt, *J* = 7.3, 0.8, 1H), 4.57 (t, *J* = 7.0 Hz, 2H), 3.18 (t, *J* = 6.9 Hz, 2H), 3.01 (s, 3H) ppm; ^{13}C NMR (100 MHz, $CDCl_3$) δ 185.6, 164.1, 162.8, 137.8, 133.6, 133.4, 132.7, 130. 3, 129.9, 128.3, 128.0, 127.7, 125.7, 125.5, 125.2, 116.3, 43.9, 28.5, 24.2 ppm; IR (KBr): ν = 2934, 1749, 1692, 1640, 1595, 1550, 1467, 1422, 1349, 1267, 1180, 980, 947, 902, 731 cm^{-1}; MS-EI: *m/z* (%) 356 (M^+, 100), 355 (98), 77 (19).

Methyl 2,3-dimethoxy-10-methyl-9,11-dioxo-6,9,10,11-tetrahydro-5*H*-pyrrolo[3',4':3,4] pyrrolo[2,1-*a*]isoquinoline-8-carboxylate (3fa)

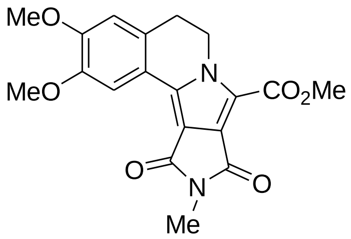

Synthesized according to the general procedure **A**; m.p.: 206-209 °C. ^{1}H NMR (400 MHz, $CDCl_3$): δ 8.17 (s, 1H), 6.71 (s, 1H), 4.66 (t, *J* = 6.9 Hz, 2H), 4.00 (s, 3H), 3.95 (s, 3H), 3.91 (s, 3H), 3.07 (s, 3H), 3.06 (t, *J* = 7.2 Hz, 2H), ppm; ^{13}C NMR (100 MHz, $CDCl_3$) δ 164.5, 162.8, 160.1, 150.6, 148.5, 133.6, 125.7, 125.5, 118.1, 117.3, 115.2, 110.4, 110.2, 56.1, 56.0, 52.2, 43.4, 27.8, 24.2 ppm; IR (KBr): ν = 3153, 3074, 2950, 1761, 1689, 1508, 1430, 1355, 1284, 1249, 1185, 1039, 987, 819, 732 cm^{-1}; MS-EI: *m/z* (%) 370 (M^{+}, 100), 355 (15).

Methyl 10-benzyl-9,11-dioxo-6,9,10,11-tetrahydro-5*H*-pyrrolo[3',4':3,4]pyrrolo[2,1-*a*] isoquinoline-8-carboxylate (3ab)

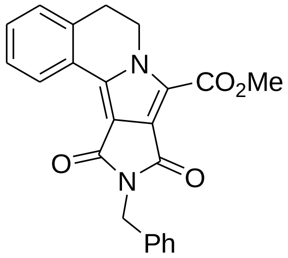

Synthesized according to the general procedure **A**; m.p.: 222-225 °C. ^{1}H NMR (400 MHz, $CDCl_3$): δ 8.49 (dd, *J* = 7.5, 1.4 Hz, 1H), 7.43 (d, *J* = 7.2 Hz, 2H), 7.39-7.27 (m, 4H), 7.24-7.20 (m, 2H), 4.76 (s, 2H), 4.69 (t, *J* = 7.0 Hz, 2H), 3.96 (s, 3H), 3.10 (t, *J* = 6.9 Hz, 2H) ppm; ^{13}C NMR (100 MHz, $CDCl_3$) δ 163.7, 162.4, 160.0, 137.1, 133.2, 132.3, 130.2, 128.54, 128.53, 127.9, 127.8, 127.6, 127.5, 125.7, 125.5, 117.7, 116.5, 52.3, 43.3, 41.9, 28.3 ppm; IR (KBr): ν = 2938, 1754, 1695, 1547, 1474, 1426, 1375, 1330, 1275, 1192, 1119, 1053, 1024, 972, 906, 738, 697 cm^{-1}; MS-EI: *m/z* (%) 386 (M^{+}, 98), 326 (41), 325 (15), 279 (16), 195 (100), 104 (34), 77 (40).

***tert*-Butyl 10-benzyl-9,11-dioxo-6,9,10,11-tetrahydro-5*H*-pyrrolo[3',4':3,4]pyrrolo[2,1-*a*] isoquinoline-8-carboxylate (3cb)**

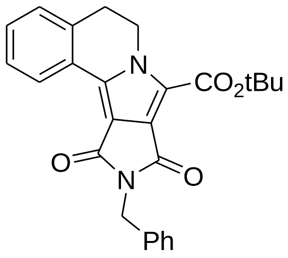

Synthesized according to the general procedure **A**; m.p.: 265-270 °C. ^{1}H NMR (400 MHz, $CDCl_3$): δ 8.50 (dd, *J* = 7.6, 1.3 Hz, 1H), 7.42 (d, *J* = 7.0 Hz, 2H), 7.37 (dt, *J* = 7.6, 1.5 Hz, 1H), 7.34-7.27 (m, 3H), 7.24-7.21 (m, 2H), 4.78 (s, 2H), 4.66 (t, *J* = 6.8 Hz, 2H), 3.10 (t, *J* = 6.9 Hz, 2H), 1.65 (s, 9H) ppm; ^{13}C NMR (100 MHz, $CDCl_3$) δ 164.0, 162.2, 159.0, 137.2, 132.6, 132.3, 130.0, 128.5, 128.3, 127.9, 127.8, 127.5, 127.4, 125.7, 125.2, 119.8, 116.3, 83.2, 43.2, 41.7, 28.4, 28.3 ppm; IR (KBr): ν = 2973, 2928, 1750, 1693, 1553, 1476, 1385, 1332, 1283, 1177, 1142, 1055, 1026, 909, 853, 745, 696 cm^{-1}; MS-EI: *m/z* (%) 428 (M^{+}, 89), 373 (24), 372 (100), 355 (20), 354 (36), 327 (25), 326 (98), 325 (24), 195 (85), 91 (15).

Methyl 10-cyclohexyl-9,11-dioxo-6,9,10,11-tetrahydro-5*H*-pyrrolo[3',4':3,4]pyrrolo[2,1-*a*] isoquinoline-8-carboxylate (3ad)

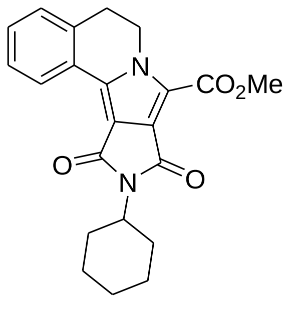

Synthesized according to the general procedure **A**; m.p.: 165-170 °C. ^{1}H NMR (600 MHz, $CDCl_3$): δ 8.53 (d, J = 7.7 Hz, 1H), 7.39 (t, J = 7.5 Hz, 1H), 7.34 (t, J = 7.4 Hz, 1H), 7.25 (d, J = 7.4 Hz, 1H), 4.71 (t, J = 6.9 Hz, 2H), 4.05 (ttt, J = 12.3, 3.8 Hz, 1H), 3.97 (s, 3H), 3.13 (t, J = 6.9 Hz, 2H), 2.22 (dq, J = 12.6, 3.3 Hz, 2H), 1.84 (d, J = 13.3 Hz, 2H), 1.71 (d, J = 11.0 Hz, 2H), 1.68 (d, J = 12.6 Hz, 1H), 1.38-1.20 (m, 3H) ppm; ^{13}C NMR (150 MHz, $CDCl_3$) 2 conformational isomers δ 164.1, 162.9, 160.2, 132.9, 132.3, 130.3, 129.9, 128.1, 128.0, 127.8, 127.3, 125.8, 125.6, 117.4, 116.7, 52.3, 52.2, 51.1, 51.0, 43.2, 30.0, 29.9, 29.84, 29.76, 28.4, 28.0, 26.3, 26.2, 26.0, 25.4, 25.3, 25.23, 25.18 ppm; IR (KBr): ν = 2937, 2855, 1746, 1696, 1546, 1427, 1340, 1280, 1198, 1036, 965, 747 cm^{-1}; MS-EI: *m/z* (%) 378 (M^{+}, 100), 319 (20), 318 (79), 297 (22), 296 (33), 265 (65), 195 (24).

General procedures B for synthesis of compounds 6

In a vial, Rose Bengal (10.2 mg, 0.01 mmol), amine **1** (0.22 mmol) and alkyne **5** (0.20 mmol) were dissolved in 1 mL acetonitrile. The reaction mixture was stirred for 24–48 hours under irradiation with 1 W green LED 30 cm strip (distance app. 5 cm) until the starting material was consumed. After the solvent was removed under reduced pressure and the crude reaction mixture was directly charged on silica gel and purified by column chromatography (hexane/EtOAc 95:5 to 9:1) to afford the corresponding product **6**.

Characterization of products 6

Dimethyl 5,6-dihydropyrrolo[2,1-*a*]isoquinoline-1,3-dicarboxylate (6aa)

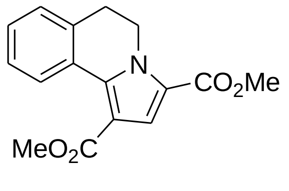

Synthesized according to the general procedure **B**, but using 2 equiv. of methyl propiolate **5a**; m.p.: 146-149 °C. ^{1}H NMR (600 MHz, $CDCl_3$): δ 8.43 (d, J = 7.8 Hz, 1H), 7.48 (s, 1H), 7.33 (t, J = 7.6 Hz, 1H), 7.29 (t, J = 7.4 Hz, 1H), 7.24 (d, J = 7.4 Hz, 1H), 4.59 (t, J = 6.6 Hz, 2H), 3.85 (s, 6H), 3.01 (t, J = 6.6 Hz, 2H) ppm; ^{13}C NMR (150 MHz, $CDCl_3$) δ 164.9, 161.3, 137.9, 134.0, 128.9, 128.5, 127.3, 127.0, 126.9, 121.5, 120.8, 112.0, 51.41, 51.39, 42.3, 29.4 ppm; IR (KBr): ν = 2949, 1700, 1536, 1453, 1428, 1254, 1169, 1082, 1012, 932, 754 cm^{-1}; MS-EI: *m/z* (%) 285 (M^{+}, 100), 254 (77).

Trimethyl 5,6-dihydropyrrolo[2,1-*a*]isoquinoline-1,2,3-tricarboxylate (6ab)

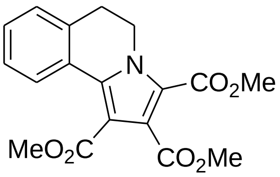

Synthesized according to the general procedure **B**; oil. ^{1}H NMR (400 MHz, $CDCl_3$): δ 8.14-8.11 (m, 1H), 7.31-7.29 (m, 2H), 7.25-7.22 (m, 2H), 4.51 (t, J = 6.6 Hz, 2H), 3.91 (s, 3H), 3.83 (s, 3H), 3.81 (s, 3H), 2.99 (t, J = 6.6 Hz, 2H) ppm; ^{13}C NMR (100 MHz, $CDCl_3$) δ 166.5, 163.8, 160.2, 136.7, 134.2, 129.3, 128.3, 127.3, 127.0, 126.8, 126.3, 118.9, 110.5, 52.6, 52.1, 51.8, 42.6, 29.3 ppm; IR (KBr): ν = 3436, 2921, 1709, 1445, 1384, 1160, 1079, 870, 776, 681 cm^{-1}; MS-EI: *m/z* (%) 343 (M$^+$, 100), 312 (68).

3-Ethyl 1,2-dimethyl 5,6-dihydropyrrolo[2,1-*a*]isoquinoline-1,2,3-tricarboxylate (6bb)[1]

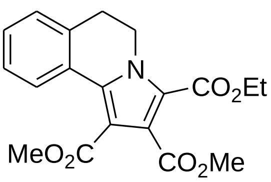

Synthesized according to the general procedure **B**. ^{1}H NMR (400 MHz, $CDCl_3$): δ 8.15-8.12 (m, 1H), 7.31-7.29 (m, 2H), 7.24-7.22 (m, 2H), 4.54-4.51 (m, 2H), 4.29 (q, J = 7.1 Hz, 2H), 3.90 (s, 3H), 3.81 (s, 3H), 2.99 (t, J = 6.6 Hz, 2H), 1.33 (t, J = 7.1 Hz, 3H) ppm; ^{13}C NMR (100 MHz, $CDCl_3$) δ 166.5, 163.8, 159.7, 136.7, 134.2, 129.3, 128.3, 127.3, 127.0, 126.7, 126.0, 119.0, 110.4, 61.0, 52.5, 51.8, 42.6, 29.3, 14.0 ppm.

Dimethyl 3-cyano-5,6-dihydropyrrolo[2,1-*a*]isoquinoline-1,2-dicarboxylate (6db)

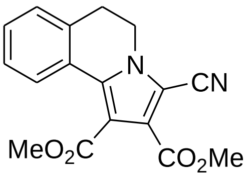

Synthesized according to the general procedure **B**; m.p.: 193-195 °C. ^{1}H NMR (600 MHz, $CDCl_3$): δ 7.65-7.63 (m, 1H), 7.32-7.30 (m, 2H), 7.28-7.26 (m, 2H), 4.24 (t, J = 6.7 Hz, 2H), 3.95 (s, 3H), 3.91 (s, 3H), 3.14 (t, J = 6.6 Hz, 2H) ppm; ^{13}C NMR (150 MHz, $CDCl_3$) δ 165.8, 161.5, 132.6, 132.3, 129.5, 128.3, 127.9, 125.7, 125.2, 122.7, 114.9, 111.3, 105.9, 52.9, 52.3, 43.5, 28.6 ppm; IR (KBr): ν = 2955, 2229, 1713, 1513, 1471, 1432, 1302, 1162, 1101, 1055, 981, 833, 774, 716 cm^{-1}; MS-EI: *m/z* (%) 310 (M$^+$, 89), 279 (100), 192 (16).

References:

(1) Zou, Y.-Q.; Lu, L.-Q.; Fu, L.; Chang, N.-J.; Rong, J.; Chen, J.-R.; Xiao, W.-J. *Angew. Chem. Int. Ed.*, **2011**, *50*, 7171.

NMR spectra of compounds 3 and 6.

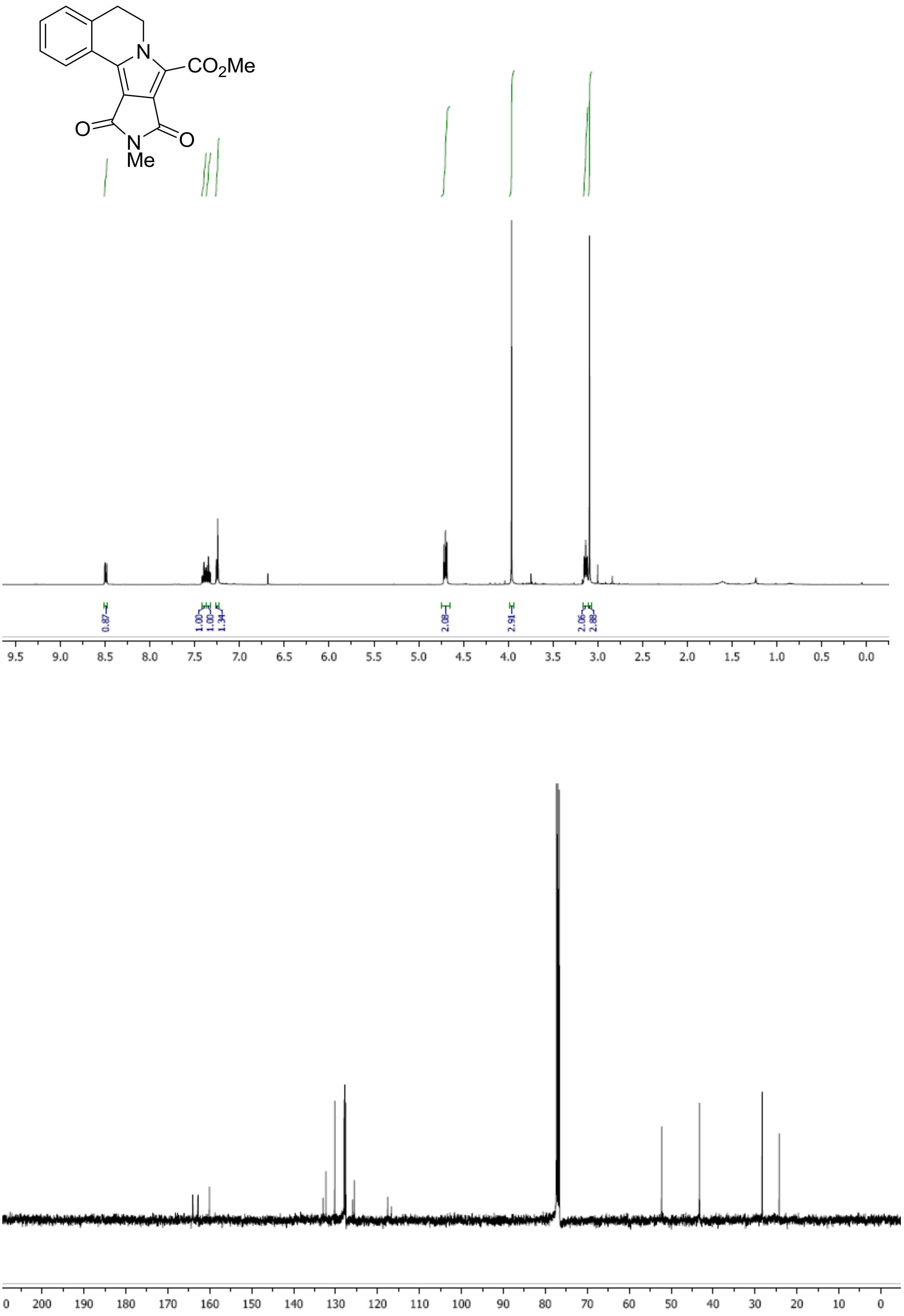

Figure S1: NMR spectra of compound **3aa**.

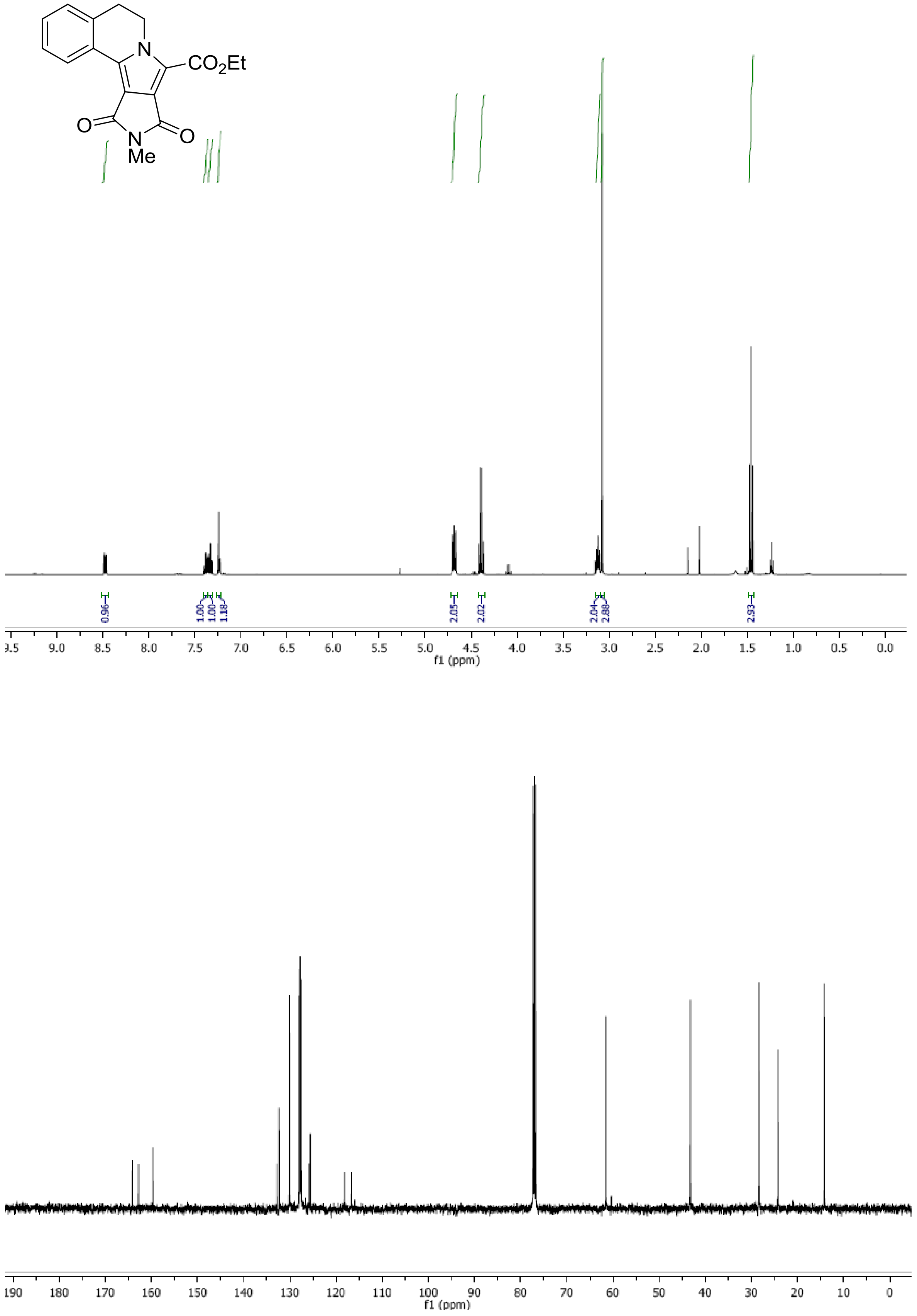

Figure S2: NMR spectra of compound **3ba**.

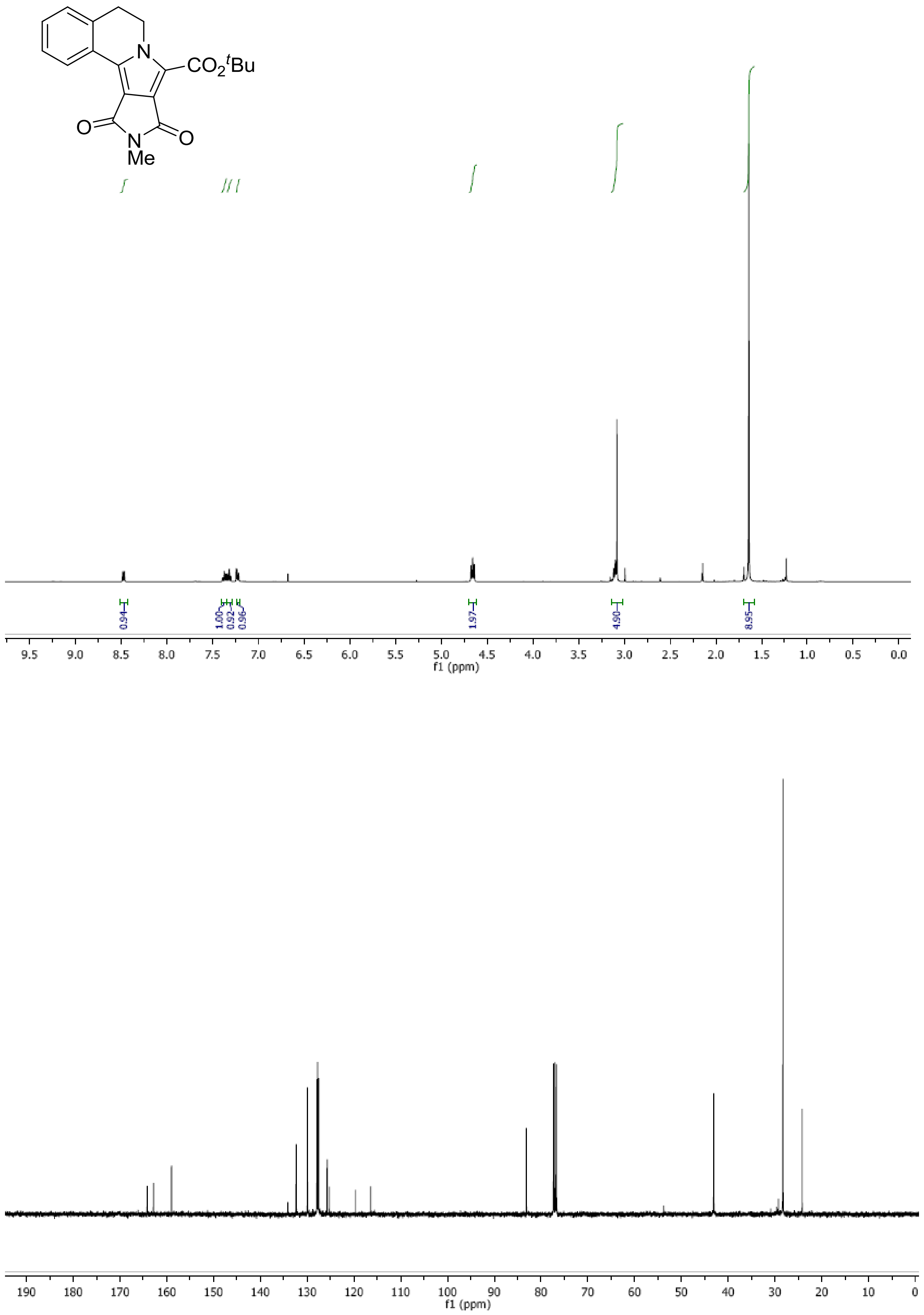

Figure S3: NMR spectra of compound **3ca**.

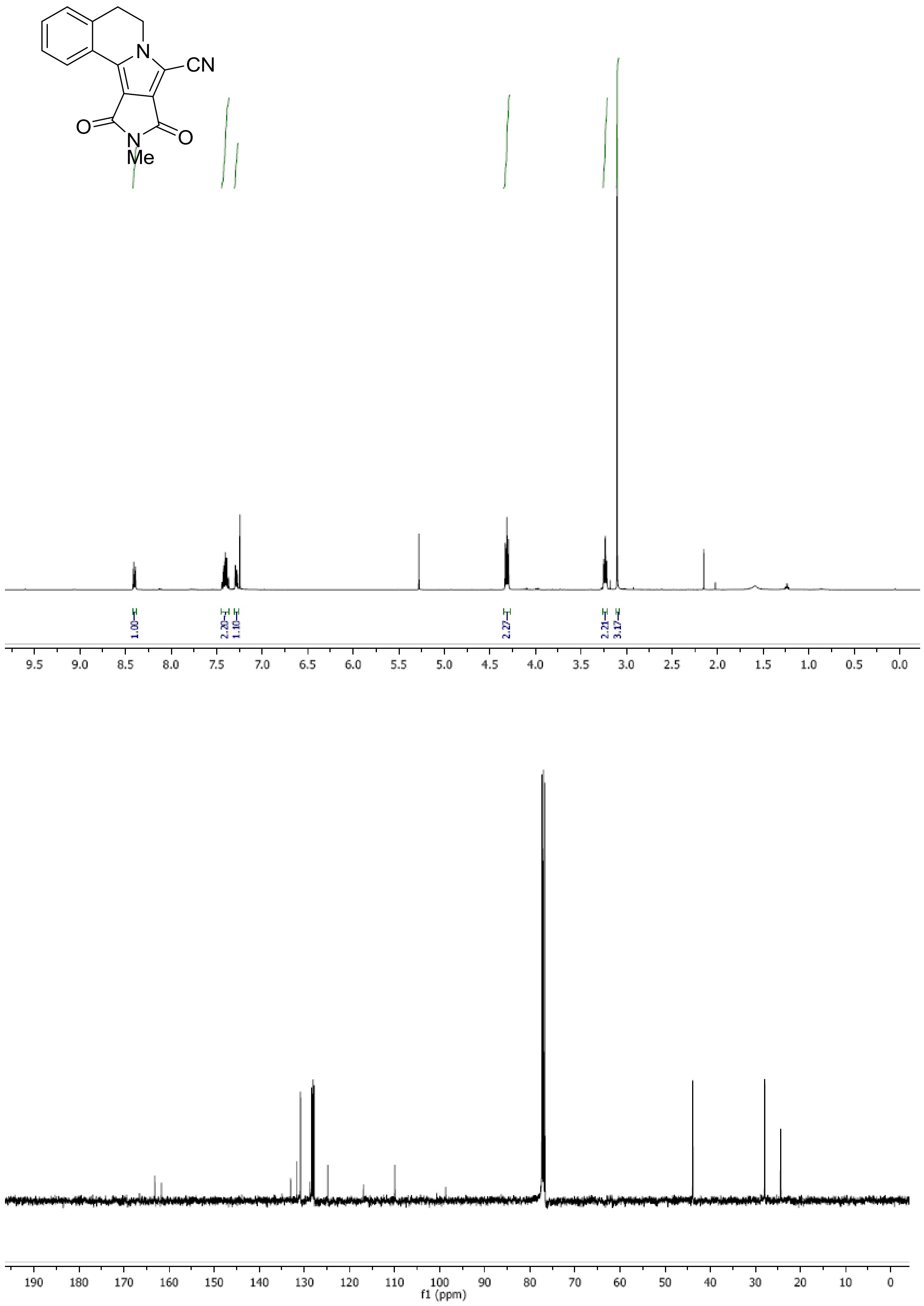

Figure S4: NMR spectra of compound **3da**.

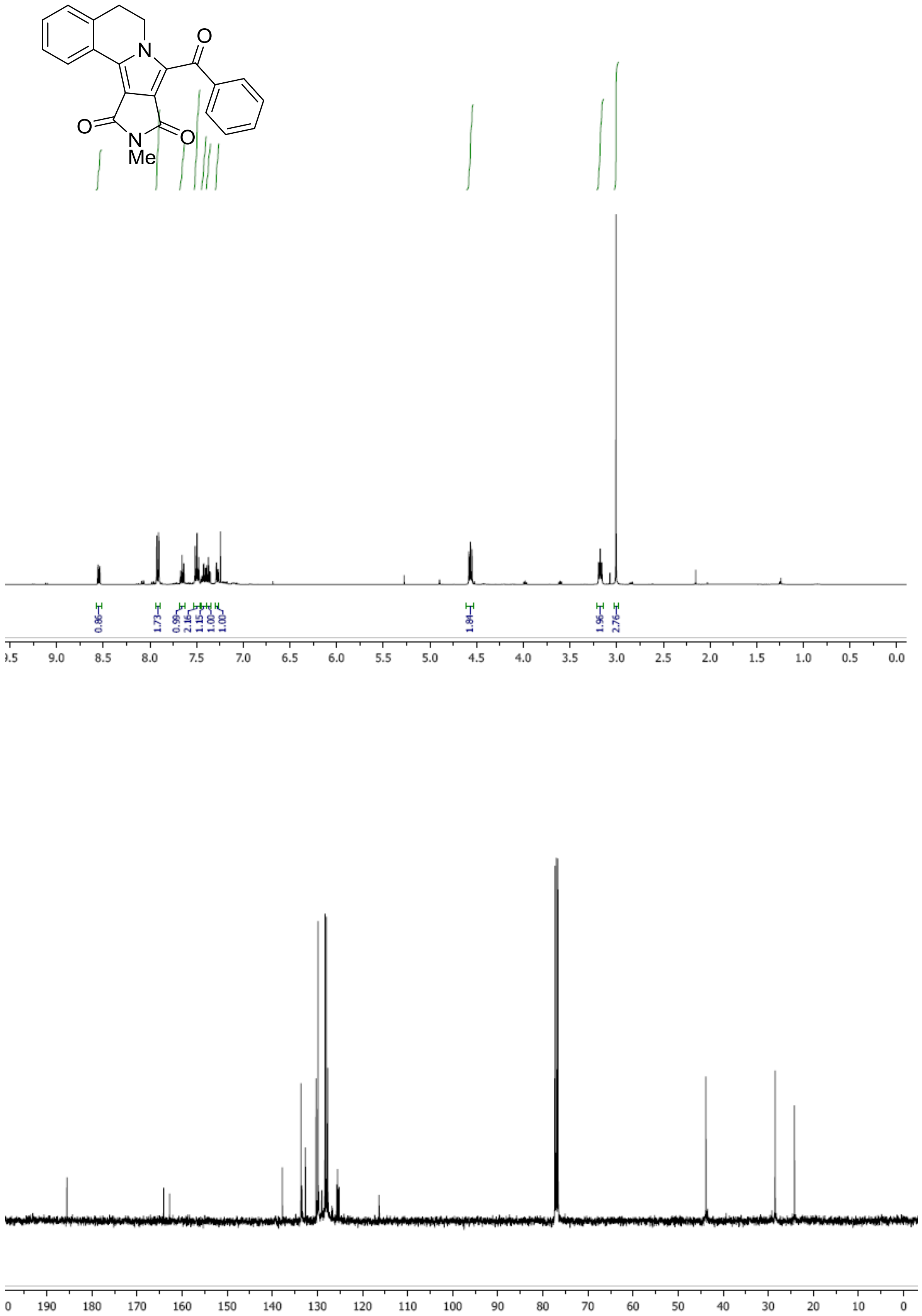

Figure S5: NMR spectra of compound **3ea**.

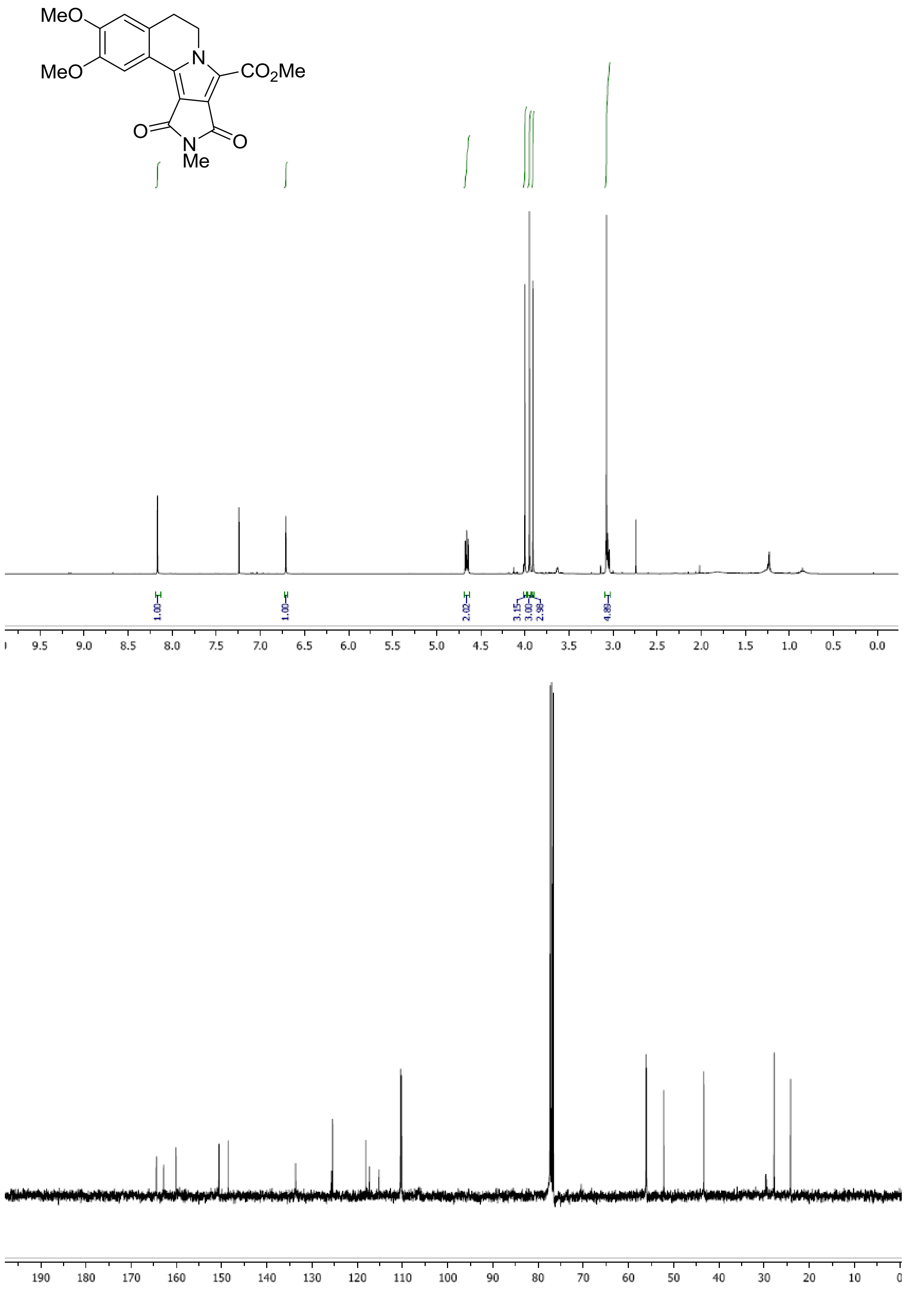

Figure S6: NMR spectra of compound **3fa**.

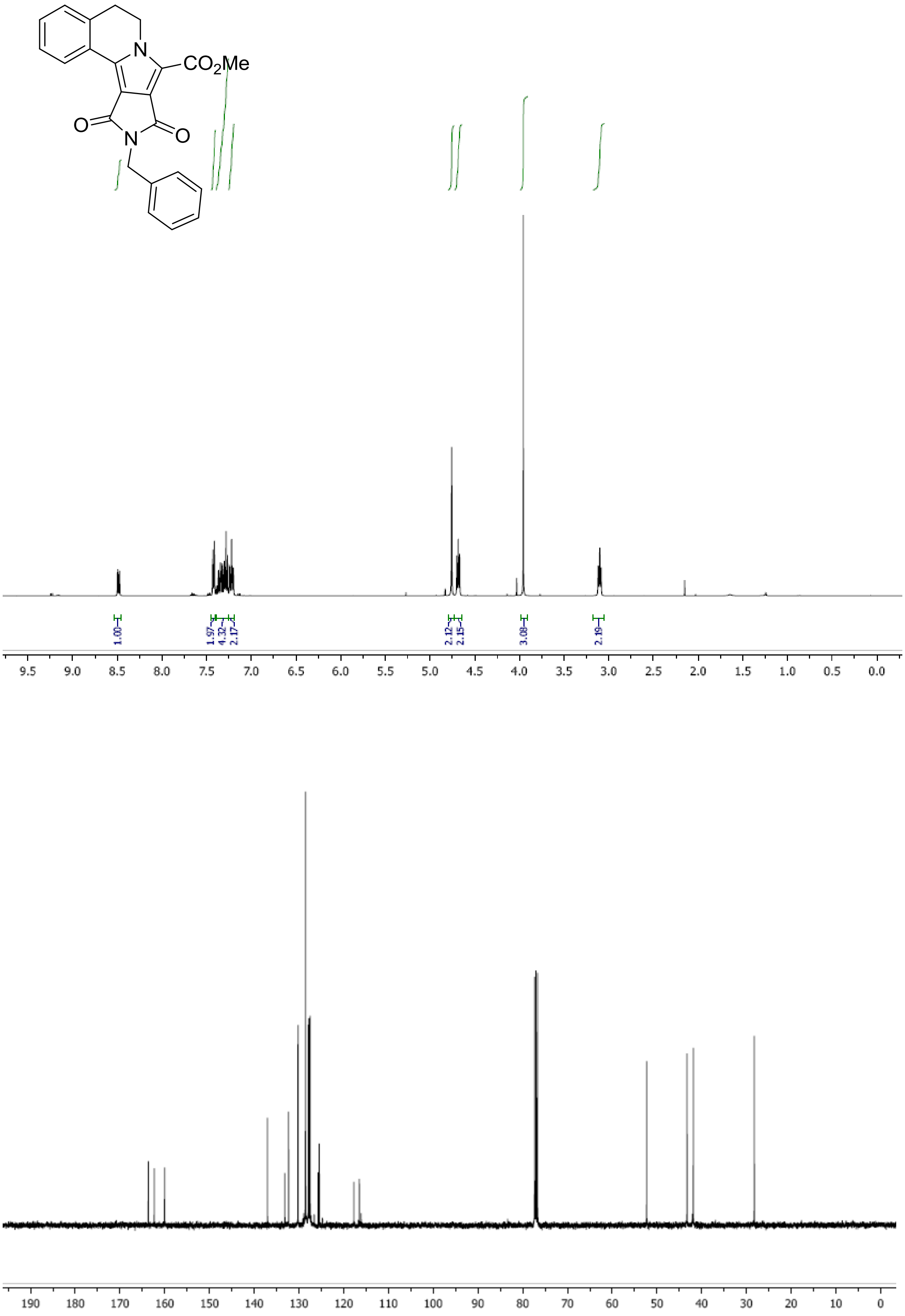

Figure S7: NMR spectra of compound **3ab**.

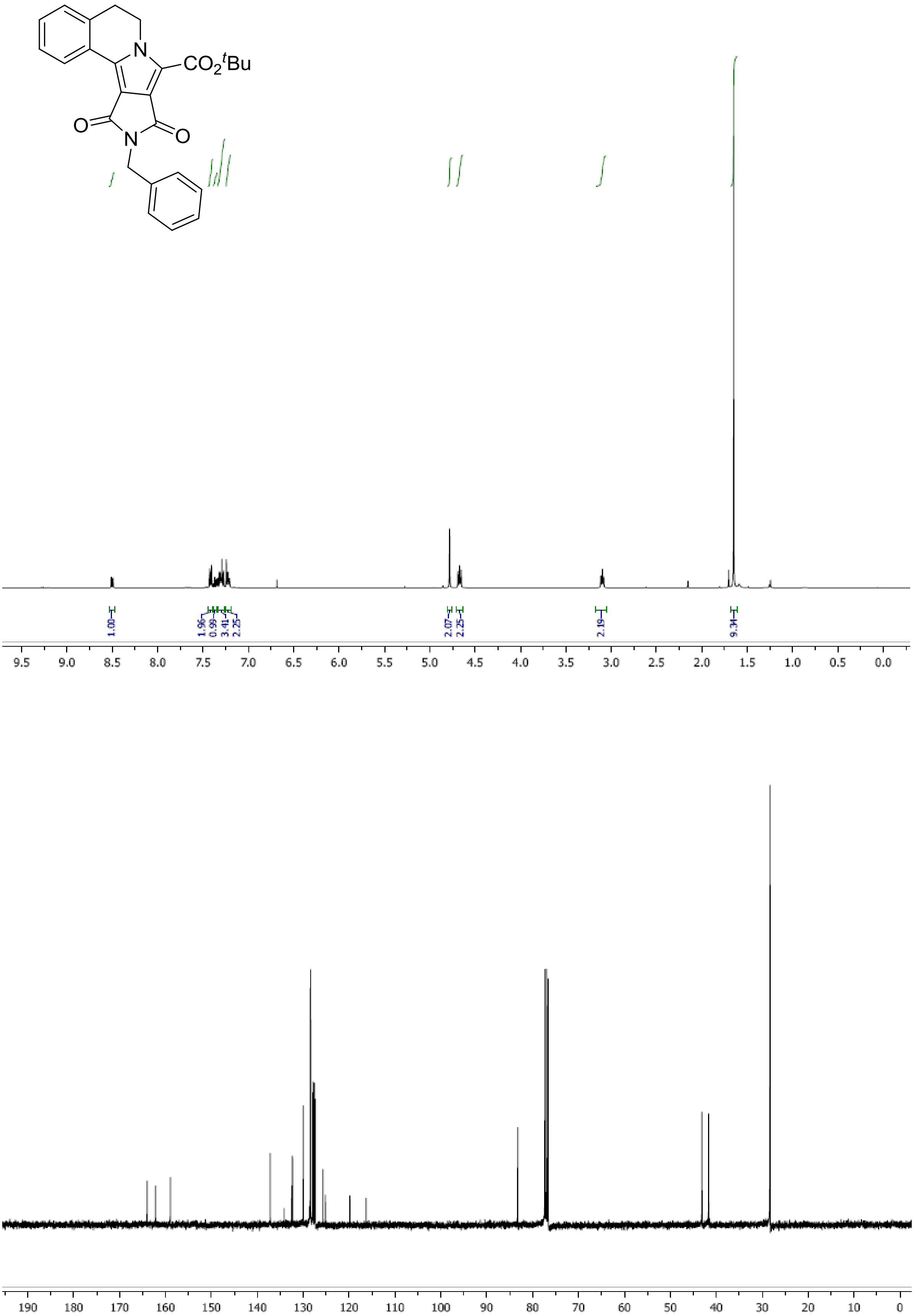

Figure S8: NMR spectra of compound **3cb**.

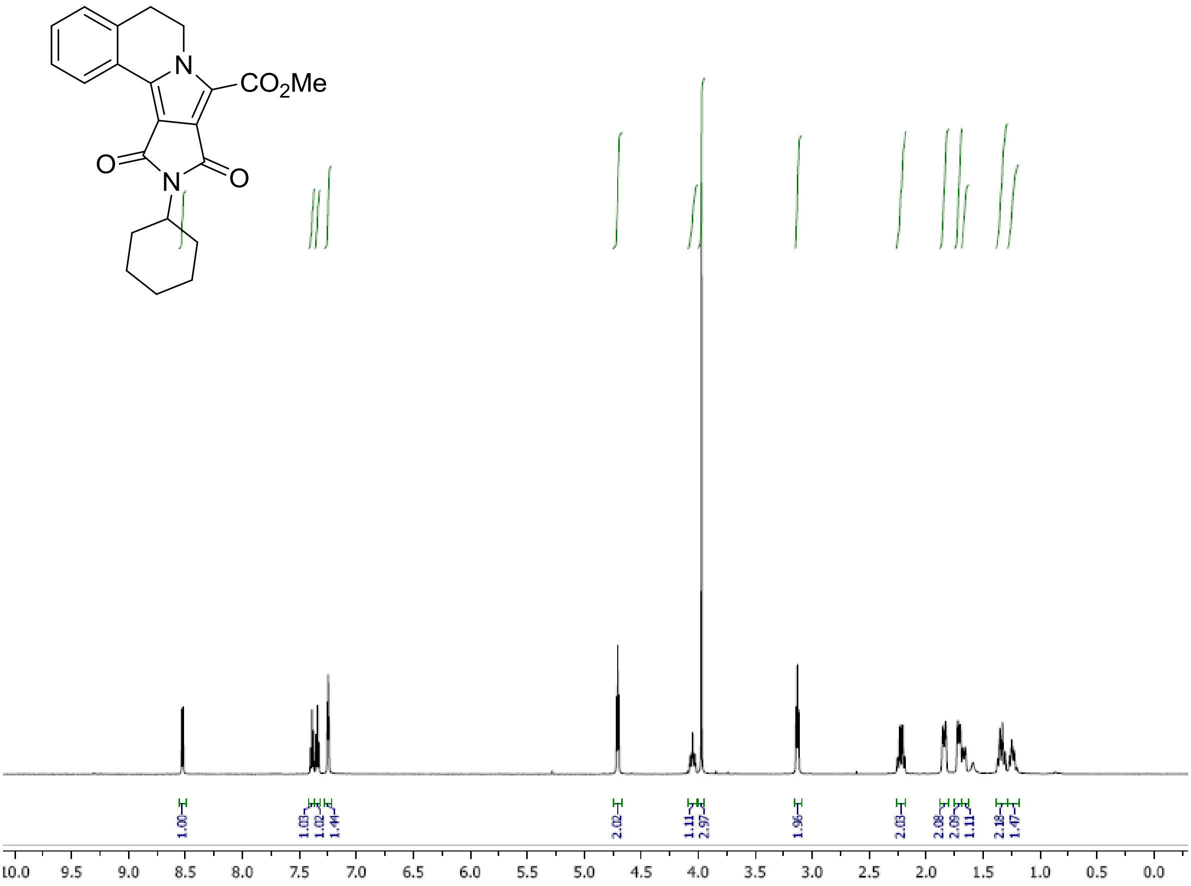

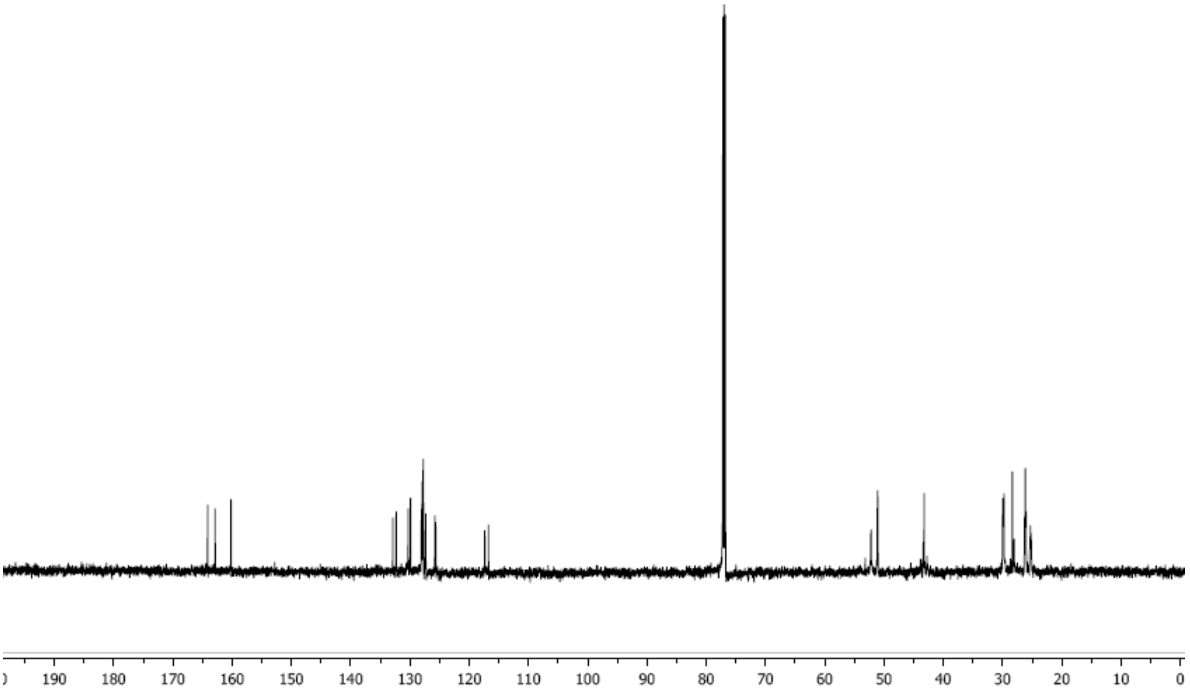

Figure S9: NMR spectra of compound **3ad**.

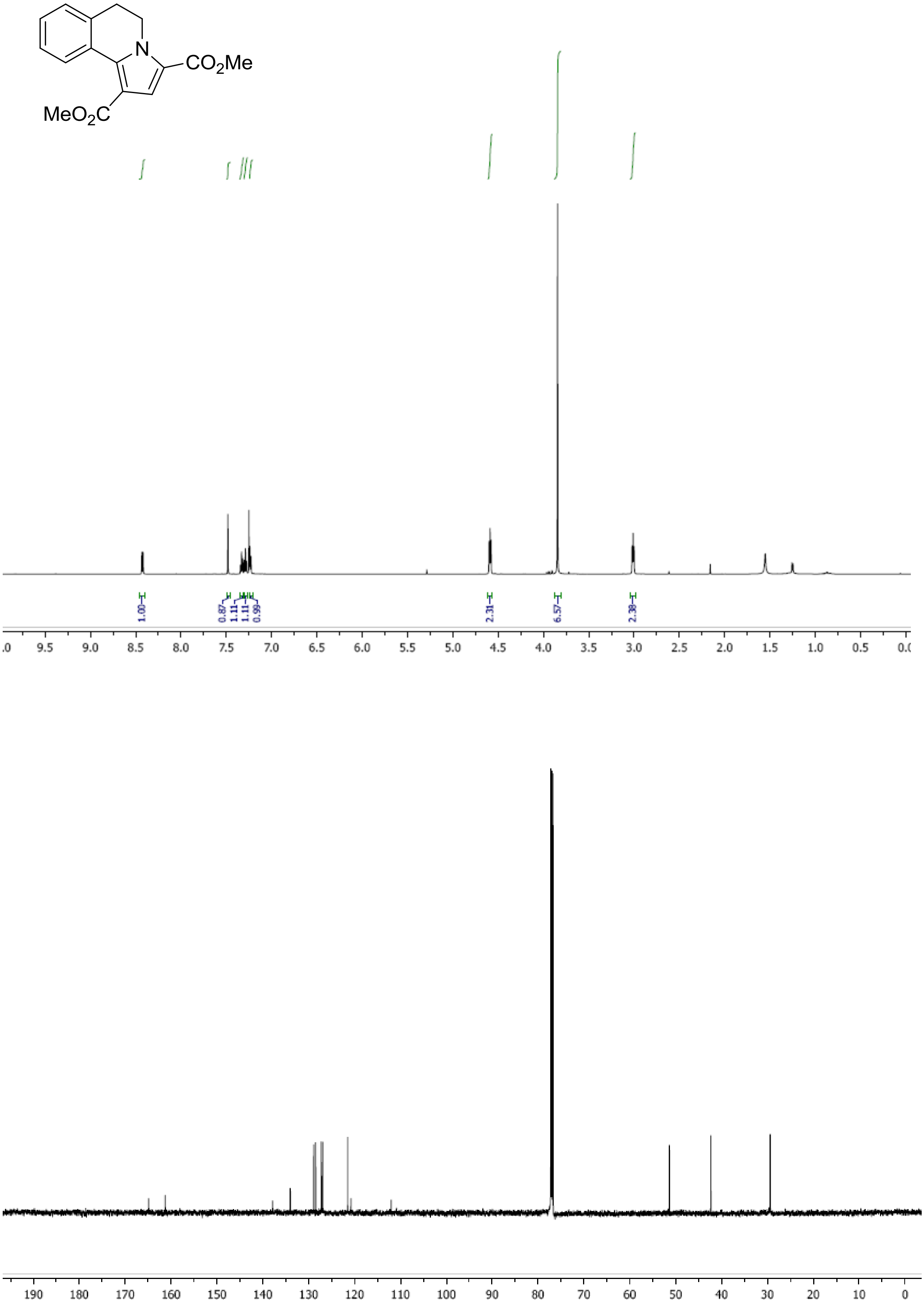

Figure S10: NMR spectra of compound **6aa**.

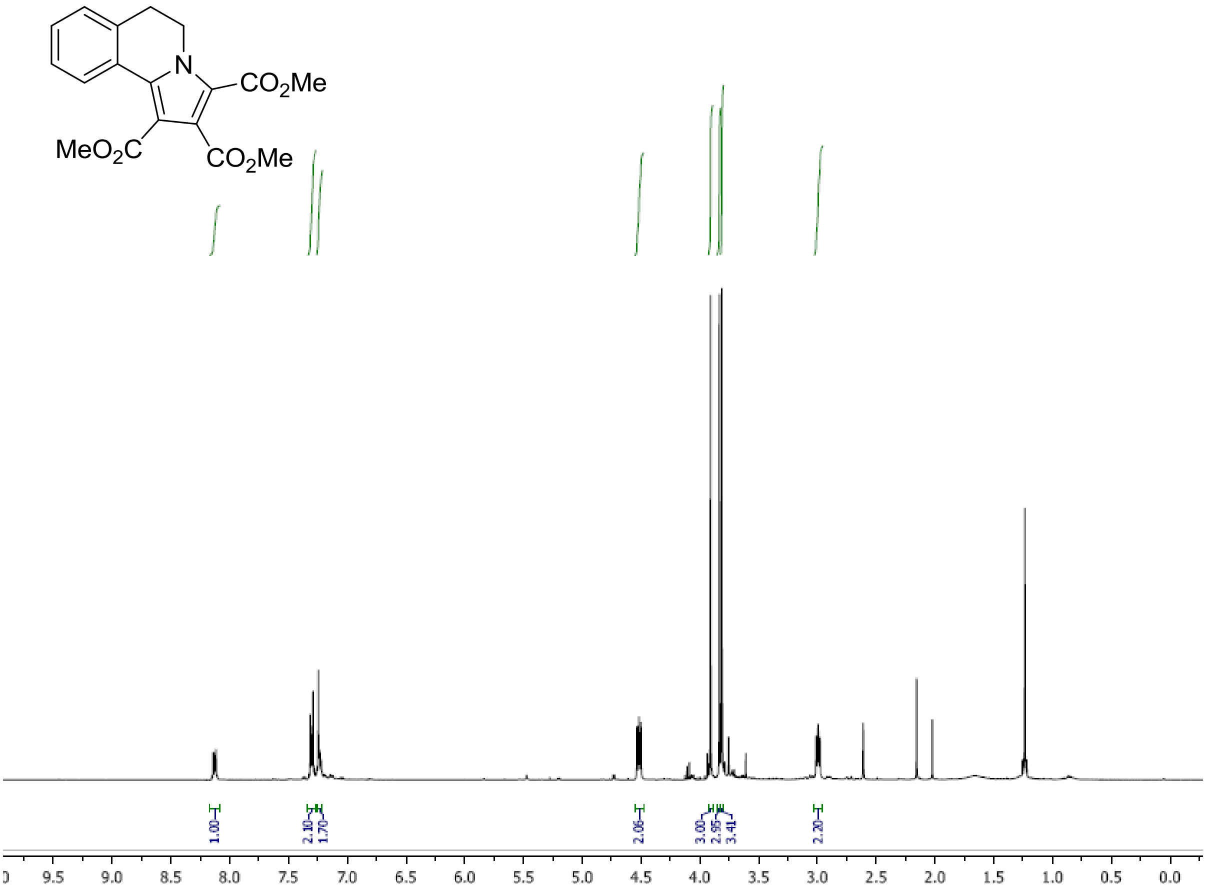

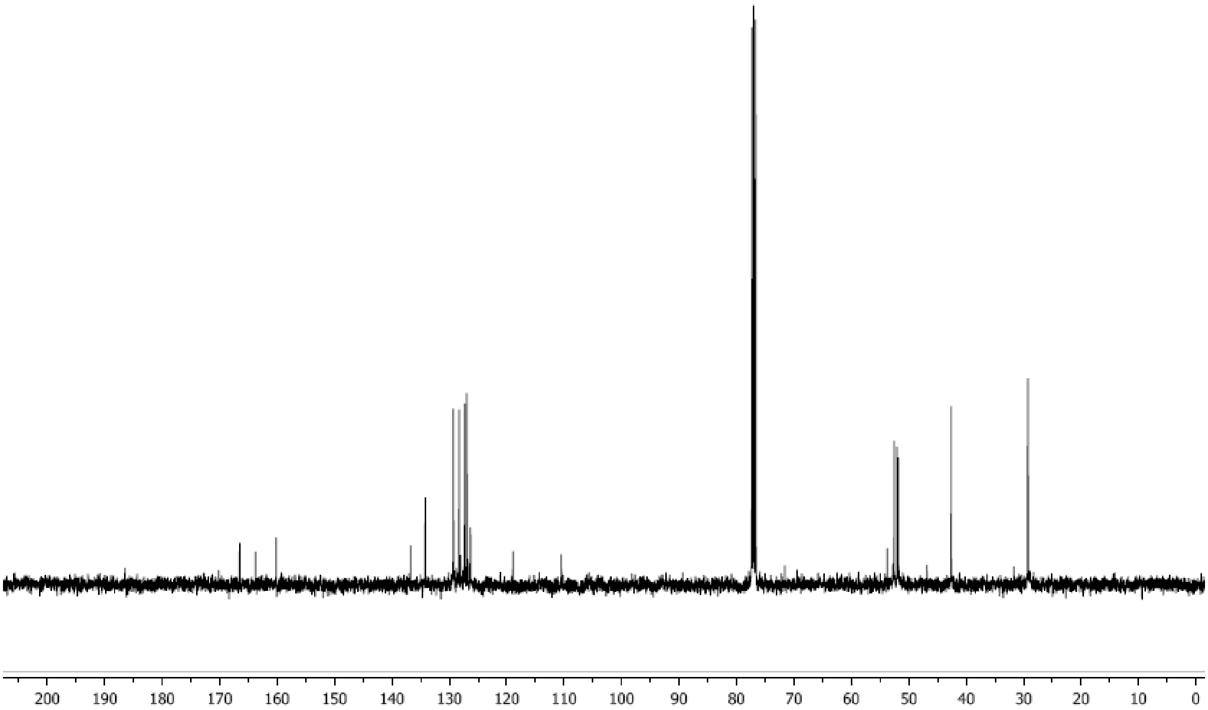

Figure S11: NMR spectra of compound **6ab**.

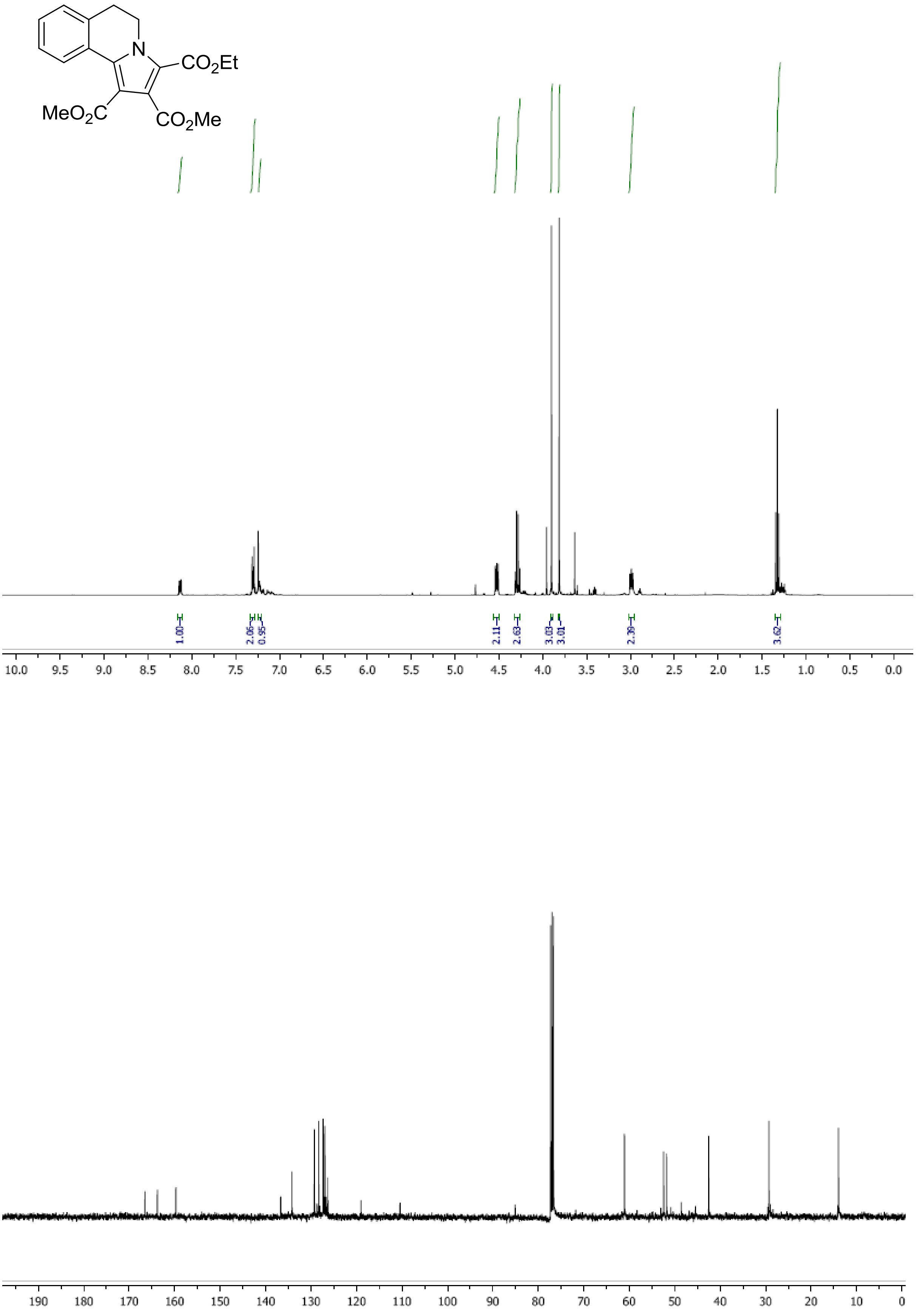

Figure S12: NMR spectra of compound **6bb**.

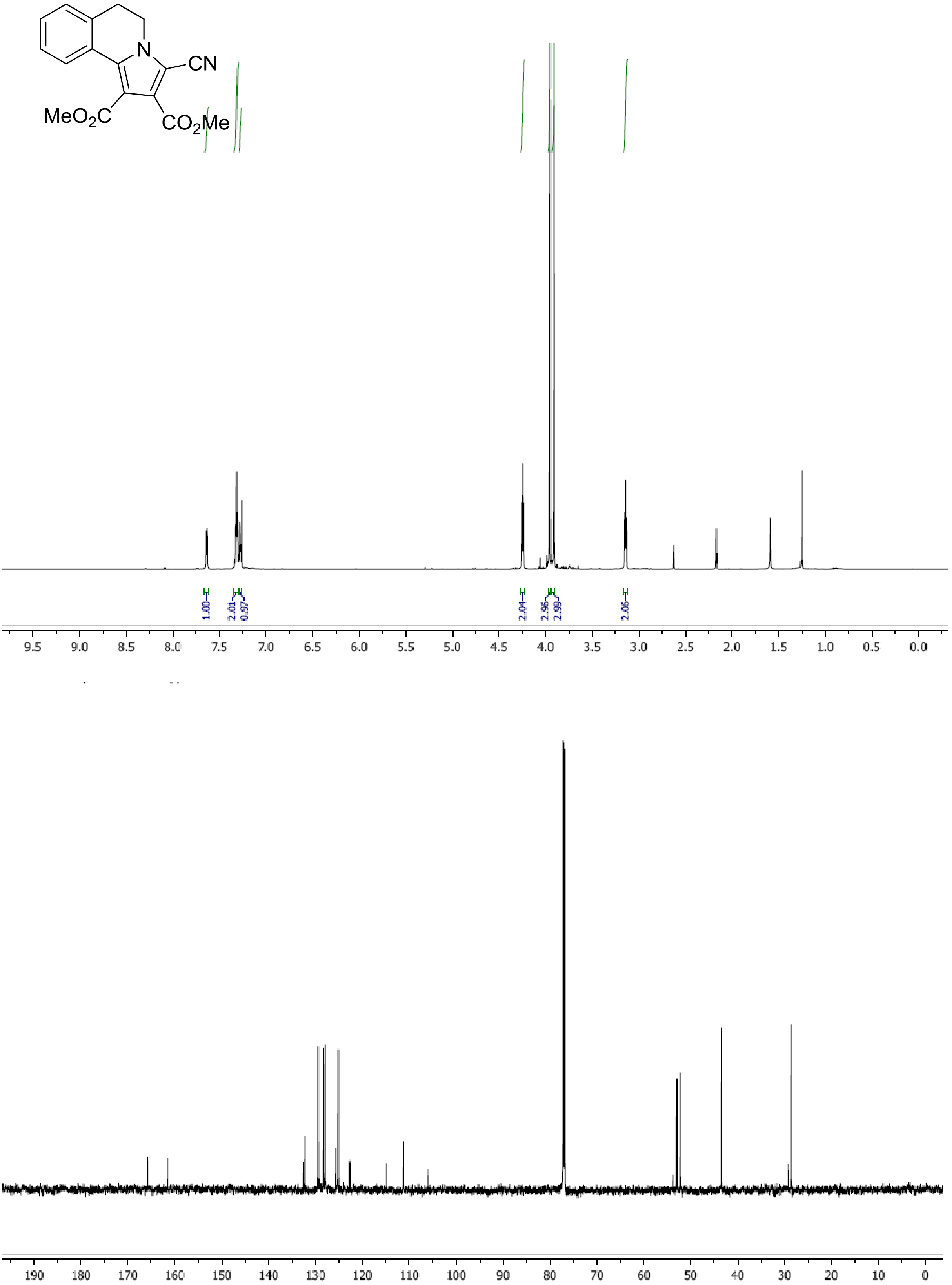

Figure S13: NMR spectra of compound **6db**.
